# Recyclable MnCl_2_-Fe_2_O_3_@CNT as Sulfur and Water-Resistant Sorbent for Gaseous Elemental Mercury Removal from Coal Combustion Flue Gas

**DOI:** 10.3390/ma18194573

**Published:** 2025-10-01

**Authors:** Zhuo Liu, Yuchi Chen, Hao Rong, Cui Jie, Xiyan Peng, Honghu Li

**Affiliations:** 1 College of Intelligent Systems Science and Engineering, Hubei Minzu University, Enshi 445000, China; 2 Hubei Novel Reactor & Green Chemical Technology Key Laboratory, Key Laboratory for Green Chemical Process of Ministry of Education, School of Chemistry and Environmental Engineering, Wuhan Institute of Technology, Wuhan 430074, China; 3 Changjiang Institute of Survey, Planning, Design and Research, Wuhan 430014, China; 4 Research Center for Environment and Health, School of Information Engineering, Zhongnan University of Economics and Law, Wuhan 430073, China

**Keywords:** carbon nanotube, MnCl_2_ modification, elemental mercury, SO_2_/H_2_O resistance

## Abstract

Mercury poses serious hazards to human health. Carbon nanotube (CNT) is a potential material for elemental mercury (Hg^0^) adsorption removal, however, it shows susceptibility to SO_2_ and H_2_O. Herein, CNT is first decorated with Fe_2_O_3_ then modified with MnCl_2_ (MnCl_2_-Fe_2_O_3_@CNT) to enhance SO_2_ and H_2_O resistance. The Hg^0^ removal performance and physical–chemical properties of samples are comprehensively studied. MnCl_2_(10)FeCNT (10 wt% MnCl_2_ content) has a high specific surface area (775.76 m^2^·g^−1^) and abundant active chlorine (35.01% Cl* content) as well as oxygen species (84.23% O_α_ content), which endows it with excellent Hg^0^ adsorption capacity (25.06 mg·g^−1^) and good SO_2_ and H_2_O resistance. Additionally, the superparamagnetic property can enable MnCl_2_(10)FeCNT to be conveniently recycled. After fifth regeneration, MnCl_2_(10)FeCNT can still achieve >90% Hg^0^ removal. The abundant active chlorine and oxygen species over MnCl_2_(10)FeCNT are responsible for Hg^0^ removal with HgCl_2_ as the primary product. This work demonstrates the enhancement of CNT’s resistance to SO_2_ and H_2_O by Fe_2_O_3_ and MnCl_2_ modification, which has potential application in flue gas mercury removal.

## 1. Introduction

Mercury (Hg) is one of the important pollutants recognized by the World Health Organization (WHO) due to its toxicity, atmospheric transport characteristics, and bioaccumulation [1,2,3,4]. As the primary anthropogenic mercury emission source [5,6], coal combustion leads to the decomposition of mercury compounds from coal and causes the emission of gaseous elemental mercury (Hg^0^) [7]. A part of Hg^0^ will be oxidized due to its interaction with the flue gas constituents and accumulate in fly ash. The resultant oxidized mercury (Hg^2+^) and particulate mercury (Hg^p^) can be efficiently removed by flue gas purification equipment currently installed in coal-fired power plants (e.g., wet scrubbers and dust removers) [8,9]. However, Hg^0^ with high volatility and low water solubility is difficult to eliminate by the above air purification equipment [10,11]. Therefore, mercury is emitted into the atmospheric environment primarily in the form of Hg^0^. Worldwide coal combustion produced 533 tons of mercury emissions in 2015 including 229 tons from East and Southeast Asia, 125 tons from South Asia and 46.5 tons from the European Union [12]. In 2015, coal combustion in China produced 73 tons of mercury emissions (54 tons Hg^0^) [13]. Hg^0^ can be transported over long distances by air, eventually deposited into natural water bodies and biotically converted into highly toxic dimethyl mercury (MeHg) [3,4]. MeHg is prone to bioaccumulate in the food chain, which can cause diseases in nervous and cardiovascular systems [4]. Aimed at controlling worldwide mercury pollution, the Minamata Convention signed by 128 countries came into effect in 2017 [14]. Hence, developing efficient mercury vapor removal technologies is critical for protecting people′s health and environmental safety.

To achieve mercury emission abatement, various methods have been proposed, such as adsorption [15,16,17], wet oxidation [18,19], catalytic oxidation [20,21,22], photochemical oxidation [23], photocatalytic oxidation [24,25], electrochemical oxidation [26], etc. Among these technologies, wet oxidation, photocatalytic oxidation, and electrochemical oxidation still suffer from the problems of a high construction and operation cost, remaining in the laboratory stage. Removal of Hg^0^ by adsorption is a more feasible, facile, and cost-effective method. Extensive studies have been conducted on the method of injecting activated carbons upstream of the dust collectors. Although such a technique holds great promise, some challenges still remain for its large-scale application. Activated carbon and other traditional carbon materials such as graphene generally show poor Hg^0^ removal activities owing to their limited surface active sites. Secondly, the carbon sorbents associated with mercury will be captured by the downstream dust remover together with fly ash. It is a challenging issue to separate the carbon sorbents from the fly ash [27]. Thirdly, SO_2_ and water vapor are typical components in flue gas, which can exert inhibitory effects on Hg^0^ removal via the mechanisms of competitive adsorption, active species consumption, etc. [28]. Without good SO_2_ and H_2_O resistance, the application value of the sorbent will be significantly reduced. In summary, highly efficient, recyclable, and sulfur as well as water-resistant sorbent for Hg^0^ capture is urgently needed.

One-dimensional carbon nanotube (CNT) with perfect hexagonal connected structures has attracted widespread attention due to its distinctive physico-chemical properties such as a high specific surface area, developed pore structure, distinct electrical characteristic, and good chemical durability [29,30]. The high surface area and advanced pore structure of CNT can promote the Hg^0^ mass transfer across the gas–solid interface and provide more anchoring sites for adsorbed Hg^0^. The excellent electrical conductivity can speed up the charge transfer between Hg^0^ and active sites over the CNT surface, thus promoting Hg^0^ oxidation. Additionally, some commonly existing defects or impurities on the CNT surface such as amorphous carbon and oxygen-containing functional group might be conducive to Hg^0^ adsorption. Despite such excellent performance, there are still the following issues to be addressed when applying carbon nanotubes to Hg^0^ removal. The primitive carbon nanotube only contains sp^2^-hybridized carbon and a small number of functional groups, which cannot supply sufficient adsorption sites for Hg^0^. The hydrophilic groups over the CNT surface such as hydrophilic open ends and oxygen-containing functional groups might boost the water vapor adsorption and exacerbate the competitive adsorption between Hg^0^ and water vapor [31]. SO_2_ as the common component in flue gas might also adsorb onto the defects of CNT and compete with Hg^0^ for the active sites. From this background, it is meaningful to further enhance the Hg^0^ adsorption on the CNT under complicated flue gas conditions. Manganese has been widely applied as a promoter to the improvement of Hg^0^ oxidation removal performance due to its multiple valence states and outstanding oxygen storage capacity [32]. For example, MnO_X_ was employed to modify carbon nanotubes for Hg^0^ oxidation removal [33]. Mn/CNT exhibited a decent Hg^0^ oxidation ability within the temperature range of 150 to 250 °C. Nevertheless, a 500 ppm SO_2_ presence would nearly result in the complete loss of the Hg^0^ removal activity of Mn/CNT due to sulfate formation. Previous studies indicate that halide modified sorbent can achieve good SO_2_ resistance and improved Hg^0^ removal capacity [34,35,36]. Therefore, this work is dedicated to the feasibility of modifying CNT by manganese chloride to enhance Hg^0^ capture performance and strengthen durability under SO_2_ and H_2_O presence.

Herein, recyclable MnCl_2_-Fe_2_O_3_@CNT was synthesized and tested for Hg^0^ capture from simulated flue gas. Fe_2_O_3_ decoration could endow CNT with magnetic property, thus facilitating the recycling of deactivated sorbent through magnetic separation [37]. A variety of experiments were applied to determine the physico-chemical properties of MnCl_2_-Fe_2_O_3_@CNT. The influences of flue gas constituents, especially for SO_2_ and H_2_O on the Hg^0^ capture ability, were systematically explored. The correlative mechanisms were further analyzed by the temperature programmed desorption of Hg (Hg-TPD), X-ray photoelectron spectroscopy (XPS), and kinetic analysis.

## 2. Experimental Section

### 2.1. Preparation of MnCl_2_-Fe_2_O_3_@CNT

Raw carbon nanotubes (single wall, ultra-high purity) were obtained from Zhongke Time Nano Technology Co., Ltd. (Chengdu, China). All the other chemical reagents (analytical grade) involved were acquired from Sinopharm Chemical Reagent Co., Ltd. (Shanghai, China). The preparation method for the recyclable MnCl_2_-Fe_2_O_3_@CNT is typically as follows: first, a stoichiometric amount of Fe(NO_3_)_3_·9H_2_O is dissolved in deionized water, and then with an addition of a desired quantity of carbon nanotubes under vigorous stirring. The resultant mixture is dried at 110 °C overnight and then calcined at 250 °C under N_2_ flow for 2 h to obtain the Fe_2_O_3_ decorated nanotubes with a Fe_2_O_3_ loading of 10 wt% (denoted as FeCNT). Second, MnCl_2_-Fe_2_O_3_@CNT samples with a targeting MnCl_2_ content of 1, 5, 10, 15 wt% are obtained by immersing FeCNT in MnCl_2_ solution of varying concentrations. The resulting product is put into an ultrasonic bath for 30 min and then dried at 110 °C overnight. The derived sample is denoted as MnCl_2_(x)FeCNT (x = 1, 5, 10, 15). CNT with a MnCl_2_ loading of 10 wt% (denoted as MnCl_2_CNT) was prepared by a similar method.

### 2.2. Characterization Methods

The characterization details are shown in the Supplementary Material.

### 2.3. Hg^0^ Removal Performance Evaluation

A laboratory-scale fix-bed quartz reactor was employed to determine the Hg^0^ removal activities of the obtained samples. The sketch map of the experimental setup is exhibited in Figure S1. Typically, a sample of around 100 mg is put into the quartz tube reactor, in which the reaction temperature is adjusted by a tubular electric furnace. The simulated coal combustion flue gas is composed of 0/5%vol. O_2_, 0/500/800 ppm NO, 0/600/1500 ppm SO_2_, 0/1/3/6%vol. H_2_O, 270 μg·m^−3^ Hg^0^, and N_2_ as balanced gas. The total flux of simulated gas is maintained at 500 mL·min^−1^, which corresponded to a gas hourly space velocity (GHSV) of around 72,000 h^−1^. With N_2_ carrying, the feed of gaseous Hg^0^ is supplied by a mercury permeation tube (VICI Metronics, Poulsbo, WA, USA) submersed in an oil bath with temperature regulating. The water vapor is supplied by a steam generator coupled with a constant flow infusion pump (FD-HGPP01, Suzhou Friend Experimental Equipment Co., Ltd., Suzhou, China). The water vapor concentration can be regulated by controlling the injection rate of deionized water with the pump. The gas pipes for Hg^0^ and water vapor are wrapped with heating tapes and heated to 110 °C to prevent their condensation. An empty bed experiment suggests that the Hg^0^ deposition on the pipe inner surface can be negligible. The gas constituents such as NO, SO_2_, O_2_, and N_2_ are acquired from the corresponding compressed gas cylinders. Mass flow meters are employed to adjust the gas concentrations. After achieving relatively stable inlet Hg^0^ concentration during 30 min, the gas flow is switched to pass through the quartz reactor for the Hg^0^ removal ability evaluation. The detailed experimental conditions for each experiment are shown in Table S1. An online mercury analysis device (QM201H, Suzhou Qing’an Instrument Co., Ltd., Suzhou, China) is utilized to monitor the inlet and outlet Hg^0^ concentrations. Before entering into the analyzer, the acid gas constituents and moisture are taken out of the gas stream by NaOH, Na_2_O_2_, and silica gel. The two equations below are used to calculate the Hg^0^ removal efficiency (*E_Hg_*, %) and the Hg^0^ adsorption capacity ($q_{t}$):

(1) $$E_{Hg}=\left(1-\frac{{{Hg}^{0}}_{out}}{{{Hg}^{0}}_{in}}\right)\times 100\%$$

(2) $$q_{t}=\frac{1}{m}\int_{0}^{t}{(Hg}_{in}^{0}-{Hg}_{out}^{0})\times F\times dt\times 10^{-3}$$

where ${Hg}_{in}^{0}$ and ${Hg}_{out}^{0}$ represent the instantaneous inlet and outlet Hg^0^ concentrations, respectively, μg·m^−3^; m is the mass of the sorbent, g; F denotes the total gas flow rate, m^3^·min^−1^; t denotes the reaction time, min; and $q_{t}\;$ denotes the mercury adsorption capacity, mg·g^−1^.

## 3. Results and Discussion

### 3.1. Physical and Chemical Properties of MnCl_2_-Fe_2_O_3_@CNT

**Figure 1:**
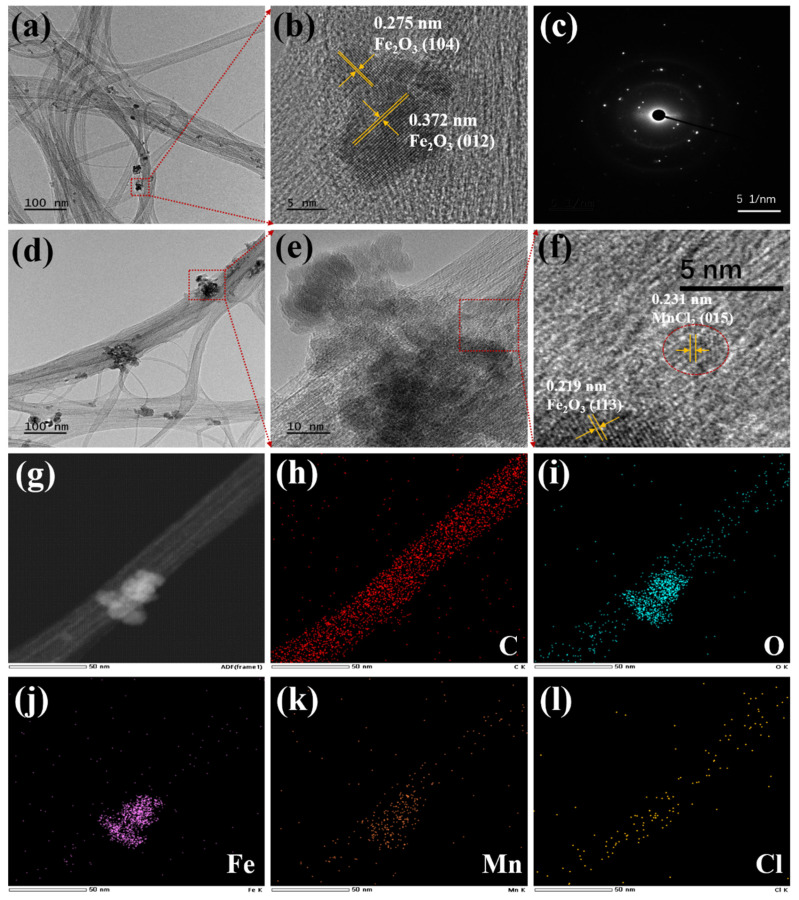
The TEM images (**a**,**b**) and SAED pattern of FeCNT (**c**); TEM images (**d**–**f**) of MnCl_2_(10)FeCNT; HAADF STEM and EDS-mapping images (**g**–**l**) of MnCl_2_(10)FeCNT.

The microstructure of the as-prepared sorbents was studied by TEM analysis as exhibited in Figure 1. In comparison with the original CNT (Figure S2), FeCNT still retains the one-dimensional (1-D) tubular structure and three-dimensional (3-D) entangled framework of CNT as shown in Figure 1a. Moreover, irregular-shaped nanoparticles with particle sizes ranging from 5 to 20 nm can be observed on the CNT surface. From the HRTEM image in Figure 1b, the interplanar lattice fringes of 0.275 and 0.372 nm can be clearly observed, which are indexed to the (104) plane and (012) plane of Fe_2_O_3_, respectively. The continuous diffraction rings in the SAED pattern as displayed in Figure 1c show the polycrystalline structure of Fe_2_O_3_ nanoparticles with relatively low crystallinity. After MnCl_2_ modification, very small particles with a size around 2.5 nm can be found on the surface of MnCl_2_(10)FeCNT as shown in Figure 1d and Figure S3. As revealed in Figure 1f, the HRTEM image exhibits a lattice spacing of 0.231 nm which belongs to the (015) plane of MnCl_2_. HAADF-STEM and EDS-mapping were further employed to explore the element distribution on the MnCl_2_(10)FeCNT surface. As shown in Figure 1g–l, the elements including C, O, Fe, Mn, and Cl are distributed on MnCl_2_(10)FeCNT in the nanoscale. The above results substantiate the existence of nano-crystalline Fe_2_O_3_ and MnCl_2_ on the tubular skeleton of the carbon nanotube, which can be further confirmed by the XRD analysis as later discussed.

**Figure 2:**
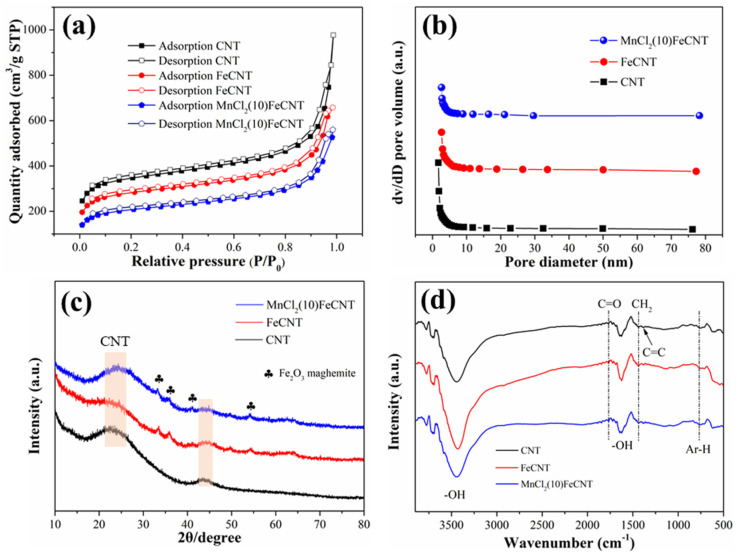
The N_2_ adsorption/desorption curves (**a**), pore size distribution curves (**b**), XRD profiles (**c**), and FTIR profiles (**d**) of the samples.

The pore structure parameters of CNT, FeCNT, and MnCl_2_(10)FeCNT are shown in Figure 2 and Table 1. The N_2_ adsorption/desorption curves for all the samples displayed in Figure 2a are consistent with the type-IV isotherm [38]. The adsorption capacity of N_2_ shows an increasing trend with the increase in relative pressure (P/P_0_). All isotherms of the samples exhibit H3 hysteresis loops at high relative pressures, suggestive of their mesoporous properties. The pore size distribution curves as displayed in Figure 2b further demonstrate the co-existence of micropores and mesopores with a pore diameter mainly smaller than 20 nm. Moreover, the BET surface area of MnCl_2_(10)FeCNT is lower than that of CNT, 775.76 m^2^·g^−1^ for MnCl_2_(10)FeCNT versus 1307.01 m^2^·g^−1^ for CNT. This is possibly owing to the fact that the introduced Mn and Fe species are deposited onto the CNT surface and partially enter into the inner pores as shown in Table 1. The lower BET surface area of MnCl_2_(10)FeCNT exhibits no correlation with its Hg^0^ removal performance, implying that the pore characteristics might not be the decisive factor for the Hg^0^ removal.

Figure 2c displays the XRD profiles of CNT, FeCNT, and MnCl_2_(10)FeCNT. For the CNT sample, the two broad peaks associated with carbon nanotube can be observed at 24° and 43.8°. For FeCNT, the diffraction peaks centered at 33.3°, 35.7°, 49.5°, and 54.2° attributed to maghemite Fe_2_O_3_ appearing in the curve [39]. This indicates that Fe_2_O_3_ is successfully decorated onto the CNT surface. No visible diffraction peaks associated with MnCl_2_ can be found in MnCl_2_(10)FeCNT, suggesting that the MnCl_2_ species is mainly amorphous or has a very small grain size.

The functional groups on the samples were characterized by the FT-IR technique. As shown in Figure 2d and Figure S4, the two broad peaks centered at 3442 cm^−1^ and 1625 cm^−1^ are attributed to symmetric/asymmetric stretching vibration and bending vibration of the -OH group, respectively [40,41,42]. The existence of C=O can be verified by the peak located at 1752 cm^−1^ [43]. Three peaks at approximately 756 cm^−1^ (aromatic hydrogen bending), 1407 cm^−1^ (C=C stretching), and 1478 cm^−1^ (-CH_2_- bending) correspond to the aromatic skeleton of CNT [38,44]. These functional groups, especially oxygen-containing functional groups, might play a certain role in Hg^0^ adsorption and oxidation. Additionally, all the samples show a similar distribution of functional groups, indicating that MnCl_2_ modification and Fe_2_O_3_ decoration will not impair the basic structure of the CNT.

**Figure 3:**
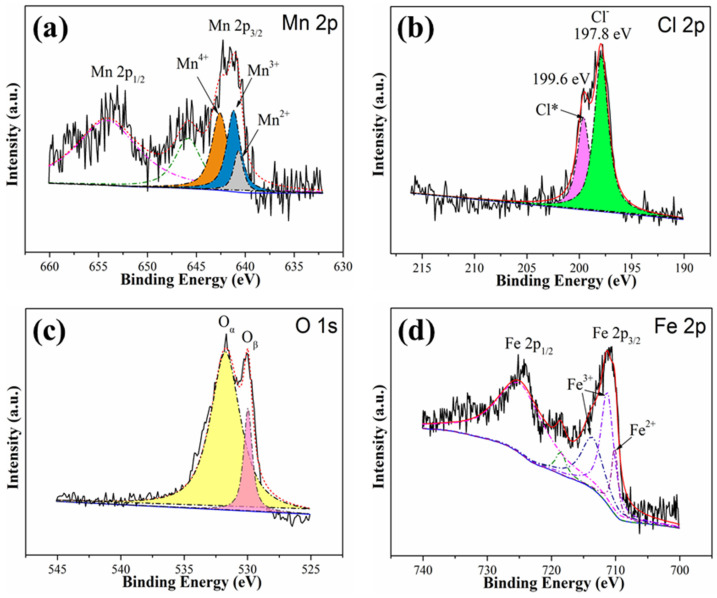
The XPS spectra pertaining to Mn 2p (**a**), Cl 2p (**b**), O 1s (**c**), and Fe 2p (**d**) of fresh MnCl_2_(10)FeCNT.

The chemical states of Mn, Cl, Fe, and O on the surface of fresh MnCl_2_(10)FeCNT were examined by XPS. For the Mn 2p_3/2_ spectra displayed in Figure 3a, three characteristic peaks located at 642.8, 641.8, and 640.7 eV are ascribed to the presence of Mn^4+^, Mn^3+^, and Mn^2+^, respectively [45,46]. It is revealed that Mn^4+^, Mn^3+^, and Mn^2+^ coexist on the surface of MnCl_2_(10)FeCNT, which might be attributed to the electronic interaction of MnCl_2_ with Fe_2_O_3_ as well as CNT support. The multiple valence states of Mn are beneficial to the electron transfer from adsorbed Hg^0^ to MnCl_2_(10)FeCNT sorbent. As shown in Figure 3b, the XPS spectra pertaining to Cl 2p are divided into two overlapping peaks located at 199.6 and 197.8 eV, which can be attributed to active chlorine species (Cl*) and ionic chlorine (Cl^−^), respectively [47,48]. Active chlorine species is considered to be active for bonding with Hg^0^ due to its high affinity to mercury. The O 1s spectra as shown in Figure 3c are resolved into two peaks centered at 529.6 and 531.5 eV, which correspond to lattice oxygen (O_β_) and chemisorbed oxygen (O_α_), respectively [49]. It can be seen that MnCl_2_(10)FeCNT possesses abundant chemisorbed oxygen species on its surface, which might be conducive to Hg^0^ adsorption. The fitted Fe 2p spectra are exhibited in Figure 3d. The binding energy peaks at 713.6 and 711.3 eV are assigned to the presence of Fe^3+^ [50,51]. The binding energy peak at around 710 eV is attributed to Fe^2+^ presence. The Fe^3+^ species is dominant on the MnCl_2_(10)FeCNT surface, which is in accordance with the XRD analysis. The EPR technique was employed to identify the vacancies over MnCl_2_(10)FeCNT. As shown in Figure S5, a signal at g = 2.003 is detected over MnCl_2_(10)FeCNT, which can be attributed to the oxygen vacancies [36].

**Figure 4:**
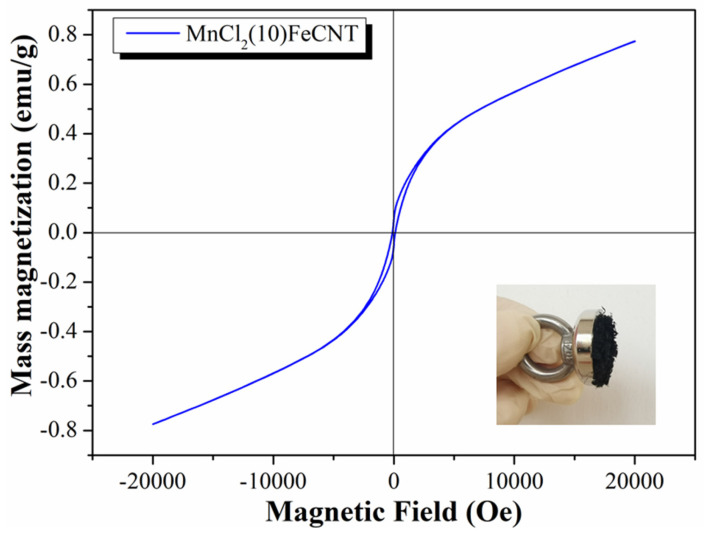
The magnetic hysteresis curve for MnCl_2_(10)FeCNT.

The magnetic hysteresis curve for as-prepared MnCl_2_(10)FeCNT is displayed in Figure 4. The fresh MnCl_2_(10)FeCNT exhibits a saturation magnetization of 0.77 emu·g^−1^. In addition, the magnetization hysteresis and coercivity for MnCl_2_(10)FeCNT are indiscernible, suggestive of its superparamagnetic property [52]. This characteristics of MnCl_2_(10)FeCNT can make it readily magnetized when it is exposed to an external magnetic field. While in the absence of the magnetic field, MnCl_2_(10)FeCNT will become unmagnetized. MnCl_2_(10)FeCNT is successfully separated from the mixture of fly ash and sorbent by a hand magnet. Thereby, the magnetically responsive MnCl_2_(10)FeCNT might be conveniently reclaimed from the fly ash by employing high gradient magnetic separators (HGMS), which increase its potential for application. The thermogravimetric (TG) analysis results as shown in Figure S6 indicate that MnCl_2_(10)FeCNT has a goo stability below 500 °C. The weight loss below 150 °C can be attributed to surface water desorption. The weight loss above 500 °C is due to the decomposition of MnCl_2_ and the carbon skeleton.

### 3.2. Hg^0^ Removal Performance of MnCl_2_-Fe_2_O_3_@CNT

#### 3.2.1. Hg^0^ Removal Performance of Different MnCl_2_-Fe_2_O_3_@CNT Samples

**Figure 5:**
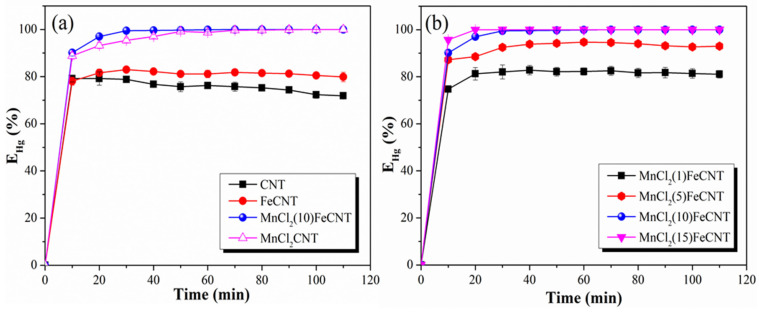
The Hg^0^ removal performances of CNT, FeCNT, and MnCl_2_(10)FeCNT (**a**); the effect of MnCl_2_ loading on Hg^0^ removal over MnCl_2_-Fe_2_O_3_@CNT (**b**).

The Hg^0^ removal abilities of different MnCl_2_-Fe_2_O_3_@CNT samples were examined under N_2_ atmosphere at 100 °C and the results are displayed in Figure 5. CNT can achieve more than 71% Hg^0^ removal during the testing period. CNT has a large specific surface area (1307.01 m^2^·g^−1^) and developed pore structure. It can facilitate the Hg^0^ diffusion across the gas–solid interface over CNT and can provide storage space for the physically adsorbed Hg^0^. Moreover, some oxygen-containing functional groups naturally existing (including C=O and COOH, etc.) on the CNT surface can function as adsorption sites for Hg^0^ and thus endow CNT with a certain chemical adsorption capacity for Hg^0^. Even so, the Hg^0^ removal efficiency of CNT substantially exhibits a decreasing trend, owing to the limited surface adsorption sites. Fe_2_O_3_ decoration slightly enhances the Hg^0^ removal efficiency over FeCNT. The introduction of Fe_2_O_3_ with certain oxidation capacity might promote the Hg^0^ oxidation over CNT [53]. For MnCl_2_(10)FeCNT, it can stably achieve almost 100% Hg^0^ removal during the testing period. The excellent Hg^0^ removal performance of MnCl_2_(10)FeCNT might be attributed to the important roles of introduced active Mn and Cl species in Hg^0^ adsorption and oxidation [54,55]. The effect of MnCl_2_ loading on Hg^0^ removal over MnCl_2_-Fe_2_O_3_@CNT was further investigated and the results are shown in Figure 5b. The 1 wt% MnCl_2_ loading results in a neglectable increase of Hg^0^ removal efficiency over MnCl_2_(1)FeCNT. For the cases of 10 wt% and 15 wt% MnCl_2_ loading, the Hg^0^ removal efficiency is increased to nearly 100%. A higher loading amount of MnCl_2_ is expected to enrich the Mn and Cl adsorption sites and thereby elevates the Hg^0^ removal performances of MnCl_2_-Fe_2_O_3_@CNT samples. If not otherwise specified, MnCl_2_(10)FeCNT is employed as the sample for further investigations in the following experiments.

#### 3.2.2. Effect of Reaction Temperature

**Figure 6:**
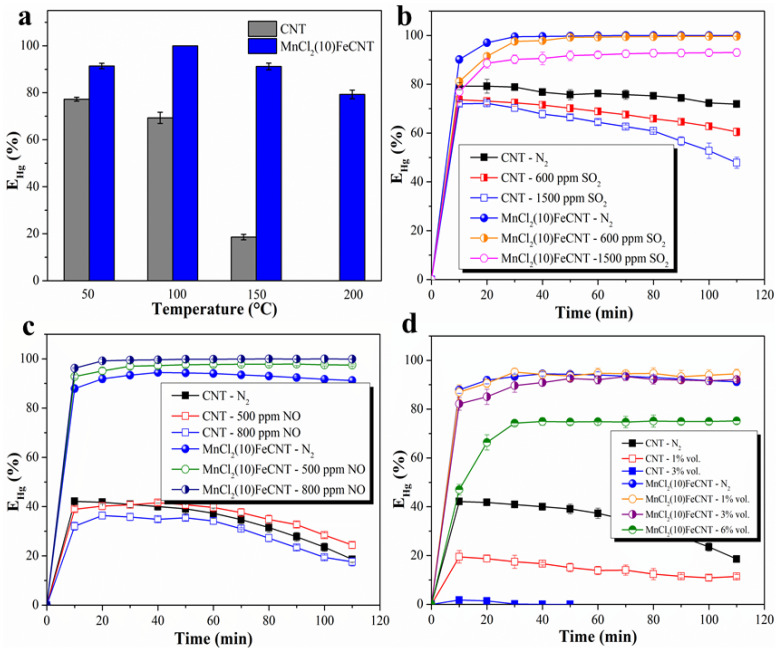
The effects of temperature (**a**), SO_2_ (**b**), NO (**c**), and H_2_O (**d**) on Hg^0^ removal over CNT and MnCl_2_(10)FeCNT.

As exhibited in Figure 6a, the Hg^0^ removal performance of MnCl_2_(10)FeCNT in the temperature range of 50–200 °C is illustrated. It is revealed that the most suitable temperature for Hg^0^ capture over MnCl_2_(10)FeCNT is 100 °C. The Hg^0^ removal efficiency increases from 91.3% to 100% when the temperature is elevated from 50 °C to 100 °C. It is suggested that chemisorption is an important path for Hg^0^ capture over MnCl_2_(10)FeCNT. A higher temperature can help the reactants to gain greater kinetic energy, thus leading to a promotive effect on the reaction of adsorbed Hg^0^ with the surface active species over MnCl_2_(10)FeCNT [56]. However, the Hg^0^ removal efficiency displays a decreased tendency with a further increase in temperature to 150 °C and 200 °C. The reason for the dropping trend of Hg^0^ removal efficiency may lie in the decomposition/desorption of the adsorbed mercury species at a higher temperature. This can be illustrated by the Hg-TPD analysis in the following discussion. Additionally, the diffusion of Hg^0^ from the gas phase onto the MnCl_2_(10)FeCNT surface will be inhibited at a higher temperature, thus resulting in a detrimental effect on the subsequent chemisorption. As for CNT, its Hg^0^ removal efficiency apparently shows a downward trend and decreases significantly in the temperature range of 50 °C to 150 °C. This result can be interpreted on the basis that most of Hg^0^ is physically adsorbed onto the CNT surface. Increasing the temperature will speed up the desorption of such weakly bonded mercury species. It can be expected that the adsorption of Hg^0^ over CNT might be severely disturbed by the commonly existing flue gas components such as SO_2_ and H_2_O due to the weak bonding of Hg^0^ to CNT.

#### 3.2.3. Effect of SO_2_

As a general acidic gas component in flue gas, SO_2_ might influence the Hg^0^ removal over MnCl_2_(10)FeCNT. The effect of SO_2_ on Hg^0^ removal was examined at 100 °C as shown in Figure 6b and Figure S7. It can be seen that SO_2_ apparently exhibits an inhibitory effect on Hg^0^ removal over CNT. When 1500 ppm SO_2_ is present in the feed gas, the Hg^0^ removal efficiency of CNT eventually decreases to 47.8% during the testing period. This observation may be attributed to SO_2_ being able to compete with Hg^0^ for the adsorption sites over CNT [28]. By way of contrast, MnCl_2_(10)FeCNT can still achieve more than 93% Hg^0^ removal with 1500 ppm SO_2_ presence. The Hg^0^ removal efficiency of MnCl_2_(10)FeCNT only decreased by around 7% with the increase of SO_2_ concentration from 0 to 1500 ppm, though MnCl_2_(10)FeCNT also faces the SO_2_ poisoning problem. The above results manifest that MnCl_2_ modification and Fe_2_O_3_ decoration can relieve the inhibitory effect from SO_2_ and greatly enhance the SO_2_ resistance of the CNT composite. Based on the previous studies [57], Cl can efficiently adsorb the Hg atom and capture its electrons due to the high electronegativity of Cl. The affinity of Cl to Hg^0^ might promote the adsorption and oxidation of Hg^0^ through the HgCl_2_ route over MnCl_2_(10)FeCNT, which is less influenced by SO_2_. Additionally, Fe species might function as a SO_2_ trap agent to protect the active species for Hg^0^ over MnCl_2_(10)FeCNT, thus enhancing its ability to resist SO_2_ [58].

**Figure 7:**
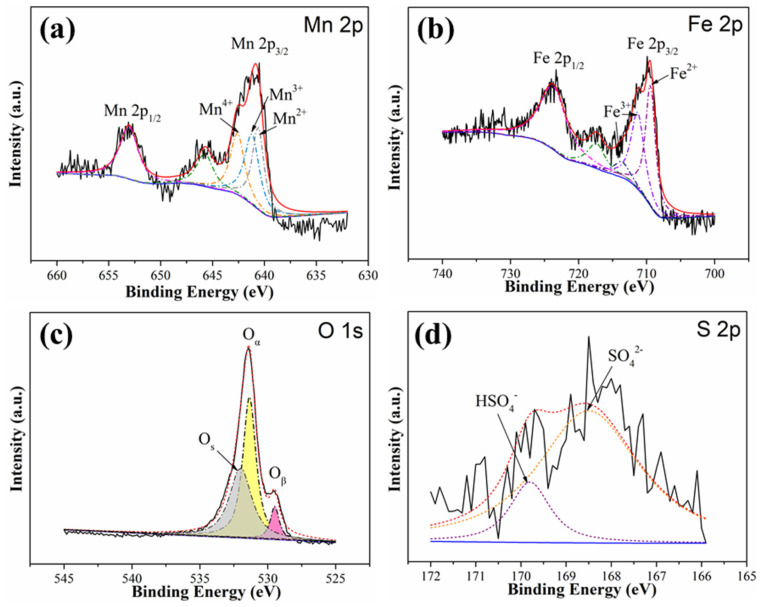
The XPS spectra pertaining to Mn 2p (**a**), Fe 2p (**b**), O 1s (**c**), and S 2p (**d**) of MnCl_2_(10)FeCNT after being used under SO_2_.

To elucidate the role of SO_2_ on Hg^0^ removal and clarify the mechanism of MnCl_2_ modification and Fe_2_O_3_ decoration on the enhancement of SO_2_ resistance, the surface chemical properties of MnCl_2_(10)FeCNT after being used under SO_2_ were further analyzed by XPS. For the Mn 2p spectra shown in Figure 7a, the intensity of the peak at about 640.7 eV attributed to Mn^2+^ is strengthened, which indicates that part of the Mn species with high valence states is reduced to Mn^2+^ due to its interaction with SO_2_. The presence of SO_2_ can cause the conversion of active Mn^4+^ and Mn^3+^ into Mn^2+^ bonded with SO_4_^2−^ that is thermally stable and chemically inert for Hg^0^ adsorption and oxidation. This will inevitably impact the Hg^0^ removal performance of the sorbent. In the Fe 2p spectra as shown in Figure 7b, the chemical state of Fe over MnCl_2_(10)FeCNT is changed due to the SO_2_ exposure. The intensity of the fitting peak at around 710 eV associated with Fe^2+^ is increased, implying that Fe^3+^ has participated in the reaction with SO_2_ and been reduced to Fe^2+^. In line with the previous studies [35,59], Fe^3+^ can compete with Mn^4+^ and Mn^3+^ for capturing SO_2_. Thereby, the Mn sites over MnCl_2_(10)FeCNT would be somewhat less influenced by SO_2_ due to the protecting effect of the Fe presence. For the O 1s spectra shown in Figure 7c, the new fitting peak appearing at 532 eV can be attributed to the presence of SO_4_^2−^ [27]. Moreover, the content of O_α_ as well as O_β_ are decreased significantly. It is confirmed that sulfate species is produced and accumulated on the MnCl_2_(10)FeCNT surface as a result of SO_2_ exposure. The formed sulfate species can also be verified by the presence of two fitting peaks at 169.9 and 168.6 eV in the S 2p spectra displayed in Figure 7d, which can be assigned to HSO_4_^−^ and SO_4_^2−^, respectively. The formation of sulfate aside, SO_2_ exposure might change the chemical state of chlorine over MnCl_2_(10)FeCNT, thus impacting its Hg^0^ removal performance. As exhibited in Figure S8, the binding energy of the shoulder peak in the Cl 2p spectra of the SO_2_-exposed MnCl_2_(10)FeCNT is decreased to 198.5 eV, lower than that of fresh MnCl_2_(10)FeCNT (199.6 eV), which indicates that the valence state of chlorine over MnCl_2_(10)FeCNT is somewhat decreased due to the SO_2_ exposure. In other words, the active chlorine species over MnCl_2_(10)FeCNT is partially consumed by SO_2_, in accordance with the previous studies [28,60]. Hence, SO_2_ can consume the active oxygen as well as chlorine species for Hg^0^ removal and cause the sulfation of the sorbent, thereby showing a negative effect on Hg^0^ removal. Thanks to the multiple active sites over MnCl_2_(10)FeCNT, it can achieve the ability to resist SO_2_ to a certain extent.

#### 3.2.4. Effect of NO

The effect of NO on Hg^0^ removal over MnCl_2_(10)FeCNT was further investigated at 150 °C as exhibited in Figure 6c. With the introduction of 500 ppm NO into the feed gas, the Hg^0^ removal efficiency of MnCl_2_(10)FeCNT is increased from 91.2% to 97.5%. A further increase in NO concentration to 800 ppm leads to a slight enhancement of Hg^0^ removal. This observation indicates that NO can facilitate the Hg^0^ removal over MnCl_2_(10)FeCNT. It has been reported that the adsorbed NO can be converted into active N-containing species such as NO_2_ and NO^+^ over metal cations with the assistance of surface active oxygen species [61,62]. This newly formed nitrogenous species might act as the new active site to oxidize Hg^0^ into Hg^2+^. This is not easy for the direct reaction of the gas-phase NO with Hg^0^, while Hg^0^ can be heterogeneously oxidized by the above discussed active nitrogenous species effectively. With regard to CNT, the introduction of 800 ppm NO into the feed gas results in a slight decrease in the Hg^0^ removal efficiency. This is possibly caused by the competitive adsorption between NO and Hg^0^. The limited active oxygen species over CNT which plays a role in Hg^0^ adsorption cannot maintain the production of active N-containing species from adsorbed NO.

**Figure 8:**
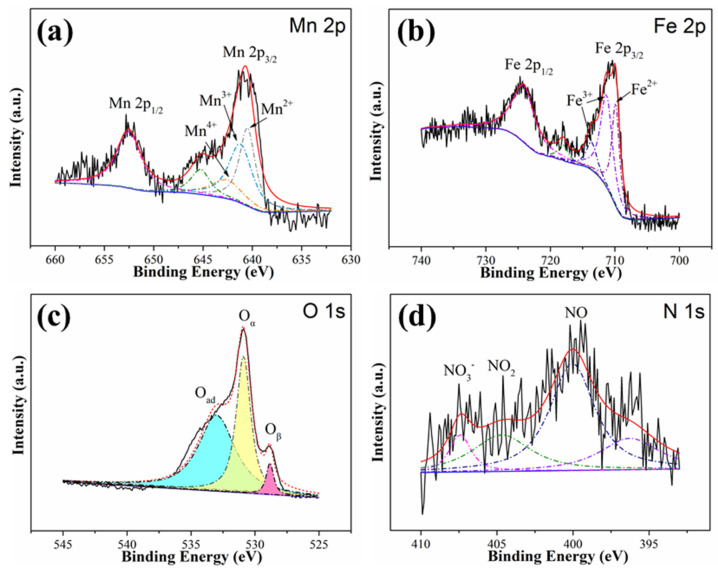
The XPS spectra pertaining to Mn 2p (**a**), Fe 2p (**b**), O1s (**c**), and N1s (**d**) of MnCl_2_(10)FeCNT used under NO.

A further clarification of the promotion effect of NO on Hg^0^ removal over MnCl_2_(10)FeCNT arises from the experiments of XPS. The XPS spectra pertaining to Mn 2p, Fe 2p, O 1s, and N 1s for MnCl_2_(10)FeCNT used under NO are shown in Figure 8. For Mn 2p spectra as exhibited in Figure 8a, the peak intensity for Mn^2+^ of MnCl_2_(10)FeCNT is strengthened after being used under NO. This observation might be owing to the conversion of Mn^4+^ and Mn^3+^ into Mn^2+^ to produce surface nitrogenous species. As shown in Figure 8b, the content of Fe^2+^ of the sample is also increased, indicating that the Fe^3+^/Fe^2+^ redox pair participates in the reaction with NO. For the O 1s spectra as exhibited in Figure 8c, a broad shoulder peak located at around 533 eV attributed to surface adsorbed oxygen species appears, while the peak intensity of O_α_ and O_β_ both decrease significantly. This result indicates that O_α_ and O_β_ have been consumed by NO to generate the aforementioned surface adsorbed oxygen species, which can be further verified by the results from the N 1s spectra as shown in Figure 8d. It can be seen that three characteristic peaks centered at 407.4, 404.9, and 400 eV associated with nitrate, surface adsorbed NO_2_ on metal sites (nitrite), and surface adsorbed NO appear in the N 1s spectra of the sample after being used under NO [63,64]. The above results confirm the formation of active N-containing species over MnCl_2_(10)FeCNT, which can act as new active sites for Hg^0^ removal. Therefore, NO exhibits a promotion effect on Hg^0^ removal over MnCl_2_(10)FeCNT.

#### 3.2.5. Effect of H_2_O

As a crucial component in the coal combustion flue gas, water vapor may strongly influence the Hg^0^ removal over MnCl_2_(10)FeCNT. The Hg^0^ removal efficiencies under different water vapor concentrations at 150 °C over the samples are displayed in Figure 6d. With the introduction of 3%vol. H_2_O into the feed gas, the Hg^0^ removal efficiency of MnCl_2_(10)FeCNT seems to decrease a little but it still maintains around 92.1%. A further increase of H_2_O content to 6%vol., and a decrease of Hg^0^ removal efficiency to 75.2% can be observed. The slight inhibitory effect of H_2_O on Hg^0^ removal over MnCl_2_(10)FeCNT can be due to the competitive adsorption of Hg^0^ and H_2_O onto the surface actives [65]. For CNT, the presence of 3%vol. H_2_O has a significant negative effect on Hg^0^ removal and decreases its efficiency to almost 0%. There are many defects on carbon nanotubes, which may result in hydrophilic groups such as oxygen-containing functional groups and hydrophilic open ends. Water molecules can easily adsorb onto the hydrophilic sites on the CNT which may be beneficial for Hg^0^ adsorption. In comparison, the decrement of Hg^0^ removal efficiency for MnCl_2_(10)FeCNT can be neglected in the presence of 3%vol. H_2_O, though MnCl_2_(10)FeCNT cannot survive the negative effect of H_2_O. Overall, MnCl_2_(10)FeCNT can achieve satisfactory H_2_O resistance. Abundant active sites are generated and exposed on the MnCl_2_(10)FeCNT surface. MnCl_2_(10)FeCNT can still afford accessible Hg^0^ adsorption sites, though part of the active sites are occupied by H_2_O. The effects of other flue gas components like NO_2_/HCl and GHSV on Hg^0^ removal have also been studied. As shown in Figure S9, both NO_2_ and HCl are beneficial for Hg^0^ removal over MnCl_2_(10)FeCNT. High GHSV will decrease the residence time for Hg^0^ removal, thus decreasing the Hg^0^ removal efficiency. In addition, the Hg^0^ removal performance of MnCl_2_(10)FeCNT under the simulated flue gas conditions was further verified aimed at its potential application. As displayed in Figure S10, MnCl_2_(10)FeCNT can achieve 91.6% Hg^0^ removal in the presence of 5%vol. O_2_, 500 ppm NO, 1500 ppm SO_2_, and 3%vol. H_2_O during the testing period. The promotional effect of O_2_ and NO might somewhat offset the impact of SO_2_ and H_2_O on Hg^0^ removal. Therefore, MnCl_2_(10)FeCNT might be a potential candidate mercury sorbent with application prospect, which can be applied in flue gas treatment due to its excellent performance and tolerance to SO_2_ and H_2_O.

#### 3.2.6. Kinetic Analysis

The previous studies have indicated the interpretation of adsorption dynamics of Hg^0^ by the pseudo-second order model, in which chemical adsorption is considered as the rate-determining step [42]. With the employment of this model, the equilibrium Hg^0^ adsorption capacity can be obtained by the experimental data. The model equation is displayed in Equation (3):

(3) $$q_{t}=\frac{t}{\frac{1}{k_{2}q_{e}^{2}}+\frac{t}{q_{e}}}$$

where $q_{t}$ and $q_{e}$ (mg·g^−1^) represent the amount of captured Hg^0^ at time t and at the equilibrium time, respectively; $k_{2}$ represents the rate constant for the pseudo-second order model, g·mg^−1^·min^−1^); and t (min) denotes the adsorption time.

**Figure 9:**
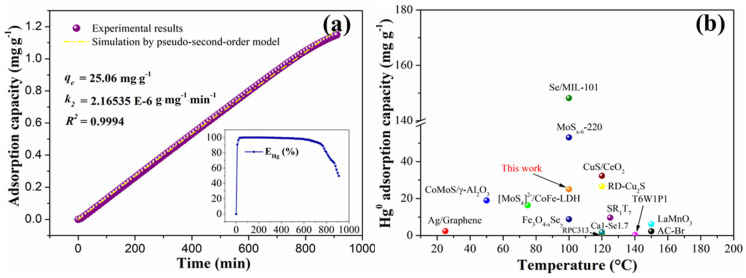
The obtained and simulated adsorption capacity curves for MnCl_2_(10)FeCNT (**a**) and the comparison of MnCl_2_(10)FeCNT with other reported sorbents (**b**).

The obtained adsorption capacity curves (according to the data displayed at the bottom right of Figure 9a) and the simulated results are shown in Figure 9a. It is shown that the pseudo-second order model is competent for describing the Hg^0^ adsorption kinetics over MnCl_2_(10)FeCNT, showing a correlation coefficient (R^2^) of 0.9994. Additionally, the adsorption capacity at equilibrium based on the kinetic fitting is 25.06 mg·g^−1^, which is one or two orders of magnitude higher than that of traditional carbon-based sorbents. It also has a Hg^0^ adsorption capacity comparable to that of the sulfide composite. The comparison of MnCl_2_(10)FeCNT with other reported sorbents is exhibited in Figure 9b (see Table S2 for the details). As can be seen, MnCl_2_(10)FeCNT outperforms most of the reported carbon-based sorbents. Moreover, the magnetically responsive MnCl_2_(10)FeCNT can be effectively separated and recycled from fly ash as shown in Figure 4. Considering its good resistance to SO_2_ and H_2_O, MnCl_2_(10)FeCNT might hold promise for future application.

#### 3.2.7. Regeneration Capability

The regeneration capability of the sorbent is critical for the real application. In this work, the mercury laden sorbent is regenerated by heat treatment at 400 °C under N_2_ flow for 2 h. As shown in Figure S11, the Hg^0^ removal efficiency of MnCl_2_(10)FeCNT does not decrease significantly after the fifth regeneration (from 100% to 92.8%). The above results reveal that MnCl_2_(10)FeCNT shows good reusability in the laboratory tests. The Mn 2p, Cl 2p, O1s, and Fe 2p spectra of MnCl_2_(10)FeCNT after fifth regeneration have been displayed in Figure S12. It can be seen that the regenerated MnCl_2_(10)FeCNT exhibits a lower content of Mn^4+^and Mn^3+^ while a larger content of Mn^2+^ than fresh MnCl_2_(10)FeCNT. The chemisorbed oxygen (O_α_) content of regenerated MnCl_2_(10)FeCNT decreases from 84.23% to 74.72% compared to the fresh MnCl_2_(10)FeCNT. The Cl* content for regenerated MnCl_2_(10)FeCNT also shows a decreasing trend. Moreover, as shown in Figure S12, the BET surface area of regenerated MnCl_2_(10)FeCNT just shows a certain decrease. This can to some extent explain the reason for the performance decline after the fifth regeneration.

### 3.3. Hg^0^ Removal Mechanism

#### 3.3.1. Hg-TPD Analysis

**Figure 10:**
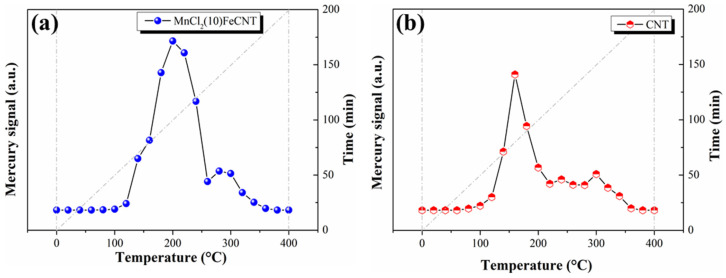
The Hg-TPD curves for MnCl_2_(10)FeCNT (**a**) and CNT (**b**).

To identify the formed mercury species on MnCl_2_(10)FeCNT, the temperature programmed desorption of Hg (Hg-TPD) from the spent MnCl_2_(10)FeCNT was carried out in comparison with the CNT. As shown in Figure 10a, the mercury desorption from MnCl_2_(10)FeCNT exhibits a strong peak at 200 °C and a weak shoulder peak at 280 °C. Previous studies indicate that the desorption temperatures for different Hg compounds can be arranged in such ascending order: HgBr_2_ < HgCl_2_ < HgS < HgO < HgSO_4_ [66,67]. Considering the chemical characteristic of MnCl_2_(10)FeCNT, the first peak can be attributed to the thermal decomposition of HgCl_2_ on the MnCl_2_(10)FeCNT surface and the latter peak can be ascribed to surface HgO. The above results manifest that the adsorbed Hg^0^ is oxidized by the active chlorine and oxygen species over the MnCl_2_(10)FeCNT surface, mainly present in the form of HgCl_2_, which is consistent with the following XPS results. For CNT, two desorption peaks located at 160 °C and 300 °C can be observed as shown in Figure 10b. The two peaks are associated with physically adsorbed or weakly bonded Hg^0^ and HgO, respectively. Physically adsorbed Hg^0^ covers a large proportion of mercury species on CNT, thus resulting in a significant decrease in its Hg^0^ removal performance with temperature increasing.

#### 3.3.2. XPS Analysis

**Figure 11:**
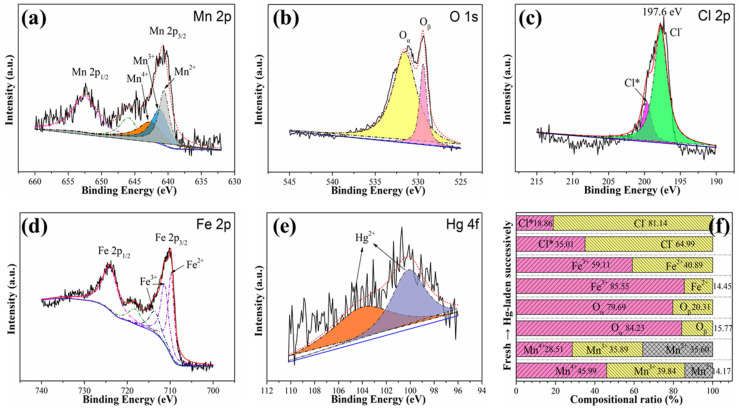
The XPS spectra pertaining to Mn 2p (**a**), O1s (**b**), Cl 2p (**c**), Fe 2p (**d**), and Hg 4f (**e**) of spent MnCl_2_(10)FeCNT after capturing Hg^0^; the comparison of chemical states between fresh and spent MnCl_2_(10)FeCNT (**f**). Cl* represents active chlorine.

To reveal the mechanism of Hg^0^ capture by MnCl_2_(10)FeCNT, the chemical states of surface elements over spent MnCl_2_(10)FeCNT after capturing Hg^0^ were further investigated by XPS analysis and the results are shown in Figure 11. As evident in Figure 11a,f, Hg-laden MnCl_2_(10)FeCNT exhibits a lower content of Mn^4+^and Mn^3+^, and a larger content of Mn^2+^ than fresh MnCl_2_(10)FeCNT, which suggests that Mn^4+^ and Mn^3+^ participate in the Hg^0^ removal process and are thereby converted into Mn^2+^. It is known that Hg^0^ is prone to adsorb onto Mn cations with high valence states, which will lead to the reduction in Mn by electron donation from adsorbed Hg^0^. Meanwhile, as shown in Figure 11b, the chemisorbed oxygen (O_α_) content of Hg-laden MnCl_2_(10)FeCNT decreases from 84.23% to 79.69% compared to the fresh MnCl_2_(10)FeCNT, accompanied with a slight increase in lattice oxygen (O_β_) content. This indicates that O_α_ is involved in the Hg^0^ capture process over the sorbent, in agreement with the previous studies [68]. Chemisorbed oxygen species including O_2_^−^ are beneficial for Hg^0^ removal and can interact with adsorbed Hg^0^ to produce HgO, thus resulting in the conversion of O_α_ to O_β_ over MnCl_2_(10)FeCNT. Figure 11c shows Cl 2p spectra for the Hg-laden MnCl_2_(10)FeCNT. It can be seen that the composition ratio of Cl* in the Cl 2p spectra obviously decreases over spent MnCl_2_(10)FeCNT accompanied with an increase in Cl^−^ content, suggestive of a conversion of Cl* to Cl^−^ involved in the Hg^0^ capture process. The consumption of Cl* and increase in Cl^−^ ratio suggests the generation of HgCl_2_ over spent MnCl_2_(10)FeCNT. Chen et al. studied the Hg^0^ removal by MnCl_2_ doped iron-carbon sorbent and found that MnCl_2_ modification enhanced the SO_2_ resistance of the sorbent [54]. The manganese sites played important roles in the Hg^0^ removal. Density functional theory (DFT) calculations in the previous studies by Ji et al. indicate that Hg^0^ can strongly interact with Cl atoms that are capable of capturing electrons on the MnCl_2_ (110) surface [55]. Both Mn and Cl are responsible for the stable Hg^0^-MnCl_2_ adsorption system with the electron transfer among Mn (d-orbit), Cl (p-, d-orbitals), and Hg (s-, d-orbitals). Xu et al. investigated the Hg^0^ removal over CuCl_2_-modified FeOCl [57]. The DFT results also indicate that the electronegativity of Cl will be enhanced due to the acceleration of electron transfer by the adjacent metal atom like Cu, thereby strengthening the electronic interaction of adsorbed Hg^0^ with Cl. By quantitative calculation and analysis as displayed in Figure 11d,f, it is revealed that the conversion of Fe^3+^ to Fe^2+^ is also involved in the Hg^0^ removal process. Fe^3+^ can capture electrons from adsorbed Hg^0^ which somewhat contribute to Hg^0^ removal over MnCl_2_(10)FeCNT. The possible mercury product over spent MnCl_2_(10)FeCNT was further examined by XPS as shown in Figure 11e. The two binding energy peaks at 104.2 and 100.7 eV in the Hg 4f spectra can be ascribed to Hg^2+^. The above results manifest that Hg^0^ is chemisorbed onto MnCl_2_(10)FeCNT and oxidized into Hg^2+^ by active chlorine (Cl*) as well as oxygen species (O*) over a sorbent surface. According to the previous studies, the abundant active chlorine and oxygen species over MnCl_2_(10)FeCNT have played important roles in the Hg^0^ removal. The involved reactions are shown as follows:

Hg^0^_(g)_ → Hg^0^_(ad)_ (4)

Hg^0^_(ad)_ + O* → HgO (5)

Hg^0^_(ad)_ + Cl* → HgCl_2_ (6)

## 4. Conclusions

In this work, recyclable MnCl_2_-Fe_2_O_3_@CNT was synthesized and tested for Hg^0^ capture from flue gas. MnCl_2_(10)FeCNT sorbent with 10 wt% MnCl_2_ content can achieve excellent Hg^0^ adsorption capacity (25.06 mg·g^−1^), which outperforms most of the reported carbon-based sorbents. SO_2_ can consume active oxygen as well as chlorine species for Hg^0^ removal and cause the sulfation of the sorbent, thereby slightly suppressing the Hg^0^ removal over MnCl_2_(10)FeCNT. In the presence of NO, the active N-containing species including adsorbed NO_2_ can be generated over MnCl_2_(10)FeCNT, which can act as new active sites for Hg^0^ removal. Therefore, NO exhibits a promotion effect on Hg^0^ removal over MnCl_2_(10)FeCNT. The presence of H_2_O slightly inhibits the Hg^0^ removal over MnCl_2_(10)FeCNT due to the competitive adsorption of H_2_O with Hg^0^ onto the active sites. In the presence of 5%vol. O_2_, 500 ppm NO, 1500 ppm SO_2_, and 3%vol. H_2_O, around 91.6% Hg^0^ removal efficiency can be achieved by MnCl_2_(10)FeCNT. Overall, Fe_2_O_3_ decoration and MnCl_2_ modification greatly promote the Hg^0^ removal performance and SO_2_ as well as H_2_O resistance of the CNT sample. Additionally, the superparamagnetic property of MnCl_2_(10)FeCNT can enable it to be conveniently separated from fly ash by magnetic separation. The Hg^0^ removal efficiency of MnCl_2_(10)FeCNT does not decrease significantly after the fifth regeneration, suggesting its good reusability. According to the Hg-TPD and XPS analysis, Hg^0^ is chemisorbed onto MnCl_2_(10)FeCNT and oxidized into Hg^2+^ mainly as HgCl_2_ by active chlorine as well as oxygen species over the sorbent surface. The abundant active chlorine and oxygen species over MnCl_2_(10)FeCNT have played important roles in the Hg^0^ removal. Although this study demonstrates the high potential of MnCl_2_(10)FeCNT for flue gas mercury treatment, the pathway to commercialization still presents exciting challenges. The next critical steps will involve engineering solutions to ensure long-term stability, conducting techno-economic analyses to evaluate cost-competitiveness, and developing scalable protocols for industrial-scale production. The effectiveness, regeneration capability, operation cost and secondary pollution risk of the proposed sorbent requires further examination under real flue gas conditions in future work.

## Figures and Tables

**Table 1:** Pore parameters of the samples.

Samples	BET Surface Area	Pore Volume	Pore Size	Micropore Area	External Surface Area
	(m^2^·g^−1^)	(cm^3^·g^−1^)	(nm)	(m^2^·g^−1^)	(m^2^·g^−1^)
CNT	1307.01	1.27	8.29	971.45	335.56
FeCNT	1065.14	0.78	7.31	790.13	275.01
MnCl_2_(10)FeCNT	775.76	0.70	8.27	560.49	215.27

## Data Availability

The original contributions presented in this study are included in the article/Supplementary Material. Further inquiries can be directed to the corresponding authors.

## Supplementary Materials

The following supporting information can be downloaded at https://www.mdpi.com/article/10.3390/ma18194573/s1, Figure S1: The schematic diagram of the experimental system; Figure S2: The TEM images of original CNT; Figure S3: The TEM images of MnCl_2_(10)FeCNT; Figure S4: The enlarged FTIR spectra for the samples; Figure S5: The EPR results of MnCl_2_(10)FeCNT; Figure S6: The TG results of MnCl_2_(10)FeCNT; Figure S7: The effects of SO_2_ on Hg^0^ removal over CNT, MnCl_2_CNT, FeCNT and MnCl_2_(10)FeCNT; Figure S8: The Cl 2p spectra of the SO_2_-exposed MnCl_2_(10)FeCNT; Figure S9: The effects of HCl and NO_2_ on Hg^0^ removal over MnCl_2_(10)FeCNT (a); The effects of GHSV and sorbent mass on Hg^0^ removal over MnCl_2_(10)FeCNT (b); Figure S10: The Hg^0^ removal performance of MnCl_2_(10)FeCNT under simulated flue gas; Figure S11: The regeneration performance of MnCl_2_(10)FeCNT; Figure S12: The XPS spectra pertaining to Mn 2p (a), Cl 2p (b), O 1s (c) and Fe 2p (d) of MnCl_2_(10)FeCNT after fifth regeneration; The N_2_ adsorption/desorption curves (e) and pore size distribution curves (f) of MnCl_2_(10)FeCNT after fifth regeneration; Table S1: The detailed experimental conditions for each experiment; Table S2: The comparison of MnCl_2_(10)FeCNT with other reported sorbents. Refs. [69,70,71,72,73,74,75,76,77,78,79,80] can be found in Supplementary Materials.
