# Supplementary material for: Recyclable MnCl2-Fe2O3@CNT as Sulfur and Water-Resistant Sorbent for Gaseous Elemental Mercury Removal from Coal Combustion Flue Gas
Source: Materials (Basel). 2025 Oct 1;18(19):4573. doi: 10.3390/ma18194573 (PMC12525843; doi:10.3390/ma18194573)
Supplement: Supplementary file 1 [file materials-18-04573-s001.zip › materials-3766154-supplementary.pdf]

Supplementary Material

Recyclable $MnCl_2$-Fe_2O_3@CNT as Sulfur and Water-Resistant Sorbent for Gaseous Elemental Mercury Removal from Coal Combustion Flue Gas

Zhuo Liu[a], *Yuchi Chen*[c], *Hao Rong*[d], *Cui Jie*[b], *Xiyan Peng*[b,*], *Honghu Li*[b,*]

a College of Intelligent Systems Science and Engineering, Hubei Minzu University, Enshi, Hubei 445000, PR China

b Research Center for Environment and Health, School of Information Engineering, Zhongnan University of Economics and Law, Wuhan, Hubei 430073, PR China

c Hubei Novel Reactor & Green Chemical Technology Key Laboratory, Key Laboratory for Green Chemical Process of Ministry of Education, School of Chemistry and Environmental Engineering, Wuhan Institute of Technology, Hubei 430074, PR China

d Changjiang Institute of Survey, Planning, Design and Research, Wuhan, Hubei 430014, PR China

*** To whom correspondence should be addressed.**

E-mail: lhhsam@outlook.com (Honghu Li), pengxiyan@stu.zuel.edu.cn (Xiyan Peng)

Characterization methods and Hg-TPD analysis

The Brunauer–Emmett–Teller (BET) specific surface area and pore characteristics of the samples were determined by N_2 isothermal adsorption at -196 °C using an ASAP2460 analyzer (Micromertics, USA). The crystal structure of different samples was investigated by the X-ray diffraction (XRD) analysis using an X-ray diffractometer (smartlab 9, Rigaku, Japan). The chemical states of the elements on the sample surface were examined by X-ray photoelectron spectroscopy (XPS), which was attained on a Thermo ESCALAB 250Xi apparatus. The Fourier transform infrared spectroscopy (FTIR) results of the samples were obtained by a Nicolet iS 10 spectrometer (Thermo Fisher Scientific, USA). Electron paramagnetic resonance (EPR) measurements were conducted on a Bruker EMXplus-6/1 (Bruker, Germany) spectrometer. The magnetic hysteresis curve of the sample was obtained by a physical property measurement system/vibrating sample magnetometer (PPMS-VSM, LakeShor, USA). The morphologies and selected area electron diffraction (SAED) patterns of the samples were acquired by a JEM-2100F (JEOL, Japan) field emission transmission electron microscope (TEM). The element distribution on the sample surface was analyzed by EDS-mapping, which was performed on a JEM-2100F instrument equipped with an X-MAX energy dispersive spectrometer (Oxford, UK). A TGA550 apparatus was employed to evaluate the thermal stability of the sample. To identify the formed mercury species on the spent sample, the temperature programmed desorption of Hg (Hg-TPD) was carried out. The Hg-laden sample in the quartz tube reactor was firstly purged by N_2 (400 ml·min^{-1}) at room temperature for 20 min. Then the sample was heated to 400 °C at a ramping rate of 2 °C min^{-1} in an N_2 atmosphere. During this process, the desorbed mercury concentration was measured by a mercury analysis device (QM201H, Suzhou Qing'an Instrument Co., Ltd).

Figure S1 The schematic diagram of the experimental system.

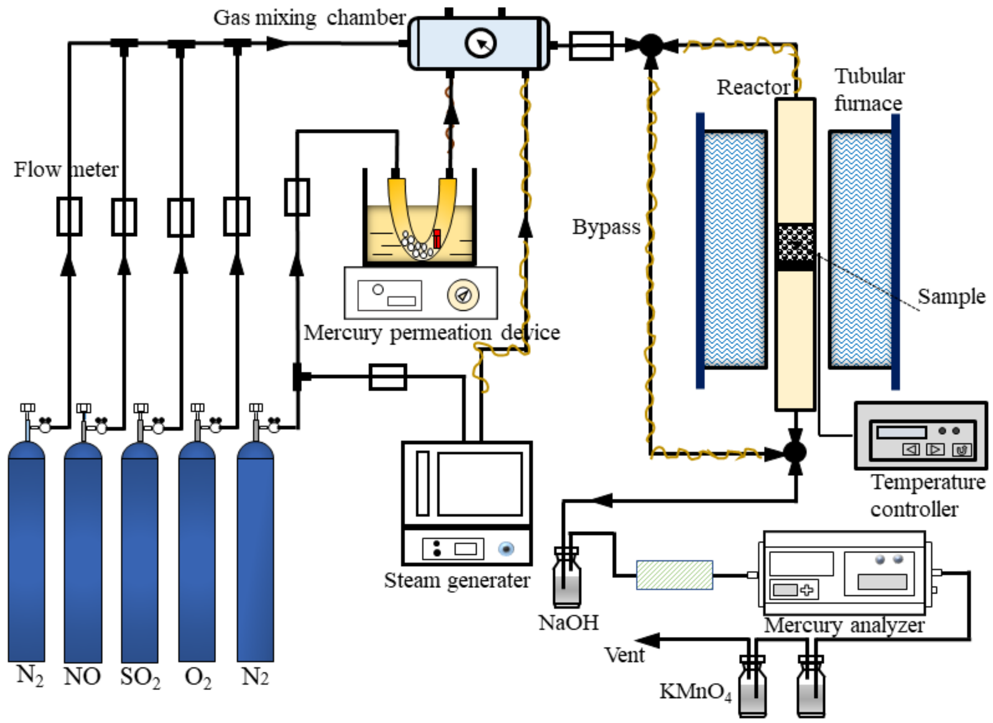

Figure S2 The TEM images of the original CNT.

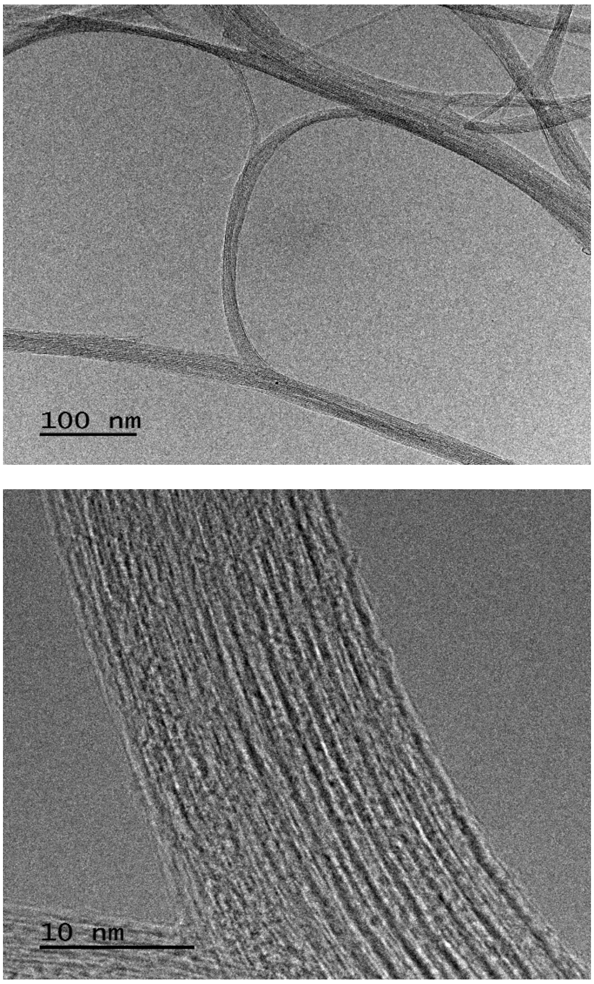

Figure S3 The TEM images of $MnCl_2$(10)FeCNT.

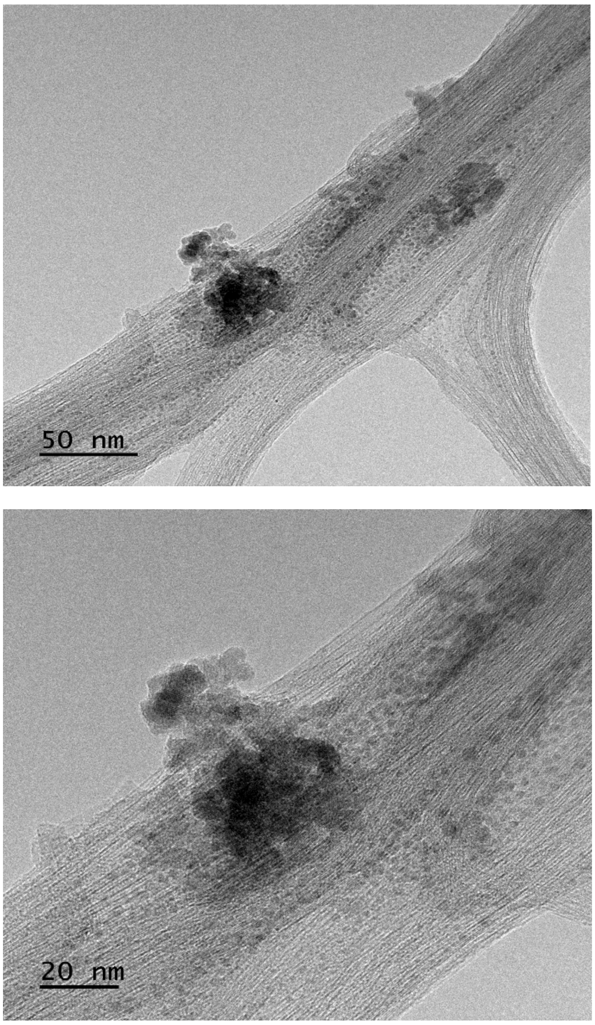

Figure S4 The enlarged FTIR spectra for the samples.

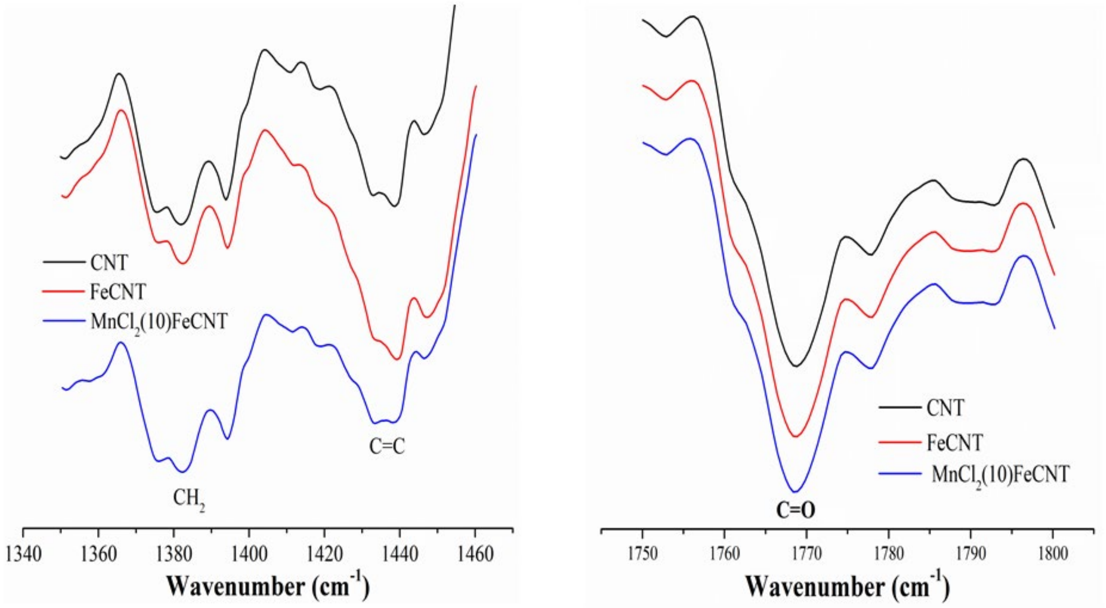

Figure S5 The EPR results of $MnCl_2$(10)FeCNT.

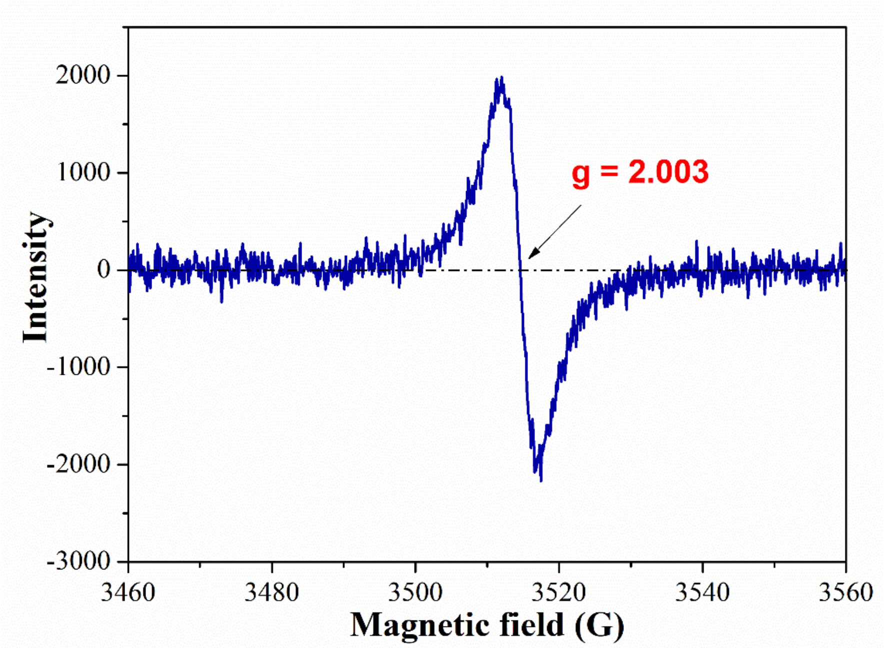

Figure S6 The TG results of $MnCl_2$(10)FeCNT.

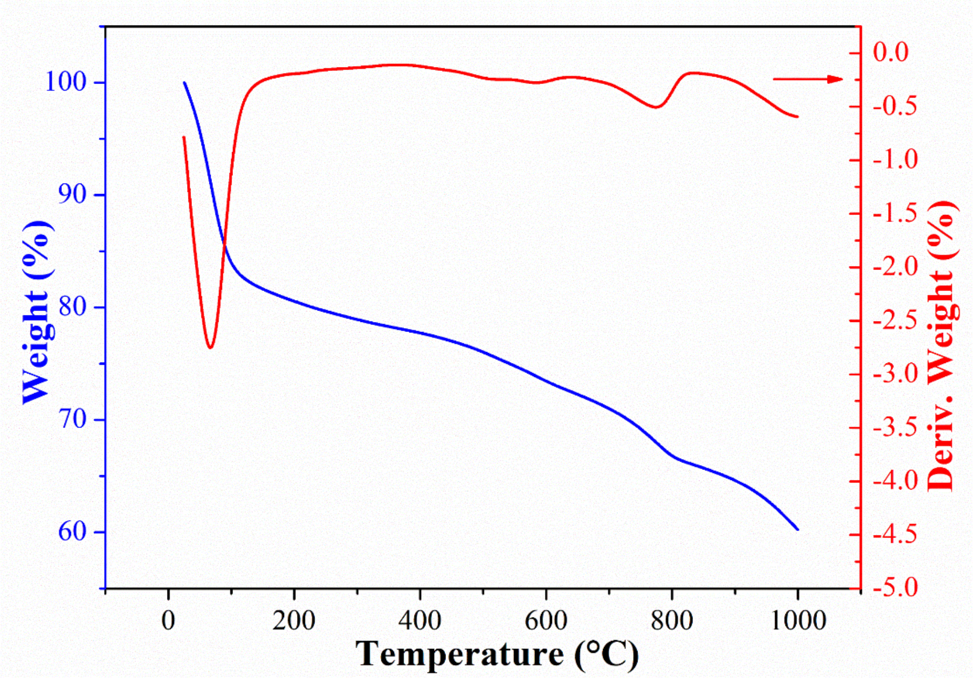

Figure S7 The effects of SO_2 on Hg^0 removal over CNT, $MnCl_2CNT$, FeCNT, and $MnCl_2(10)FeCNT$.

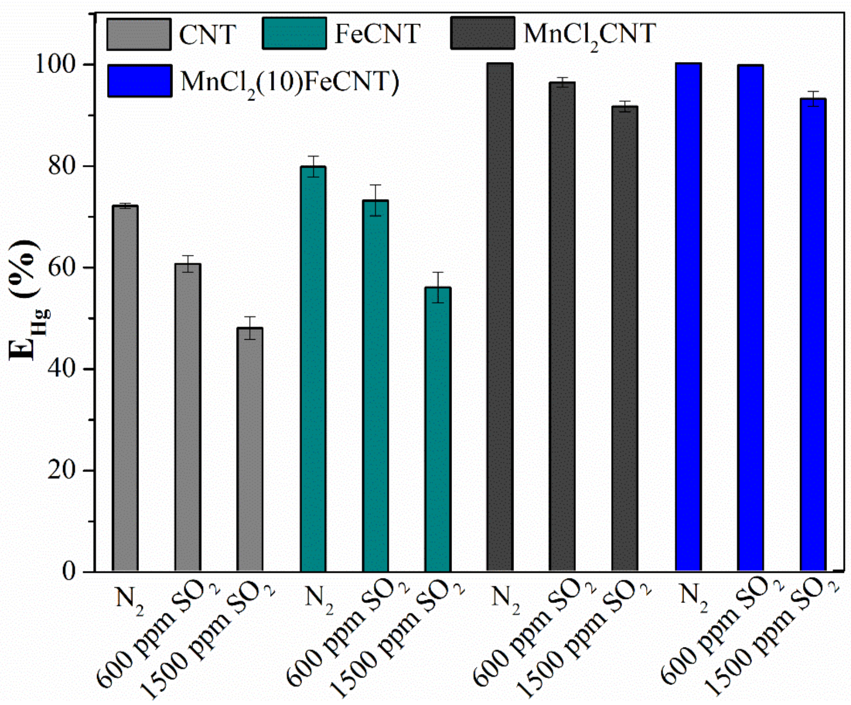

Figure S8 The Cl 2p spectra of the SO_2-exposed $MnCl_2$(10)FeCNT.

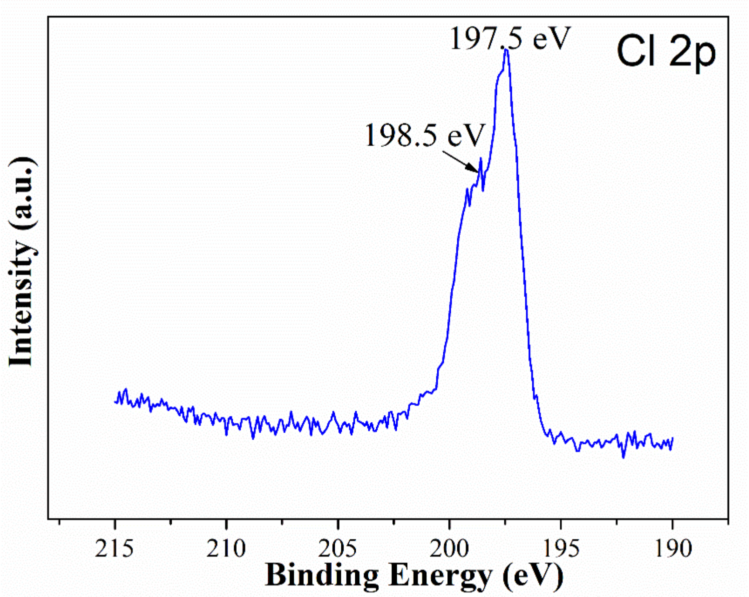

Figure S9 The effects of HCl and NO_2 on Hg^0 removal over $MnCl_2(10)FeCNT$ (a); the effects of GHSV and sorbent mass on Hg^0 removal over $MnCl_2(10)FeCNT$ (b).

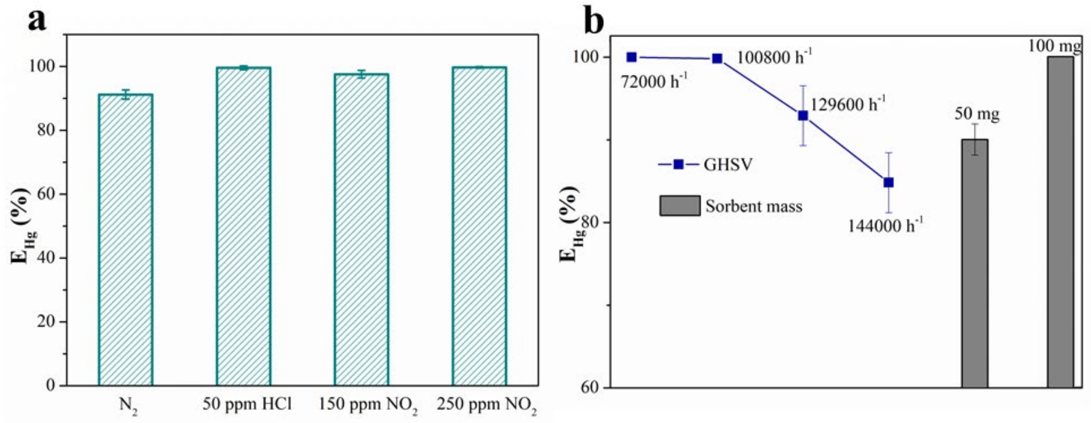

Figure S10 The Hg^0 removal performance of $MnCl_2$(10)FeCNT under simulated flue gas.

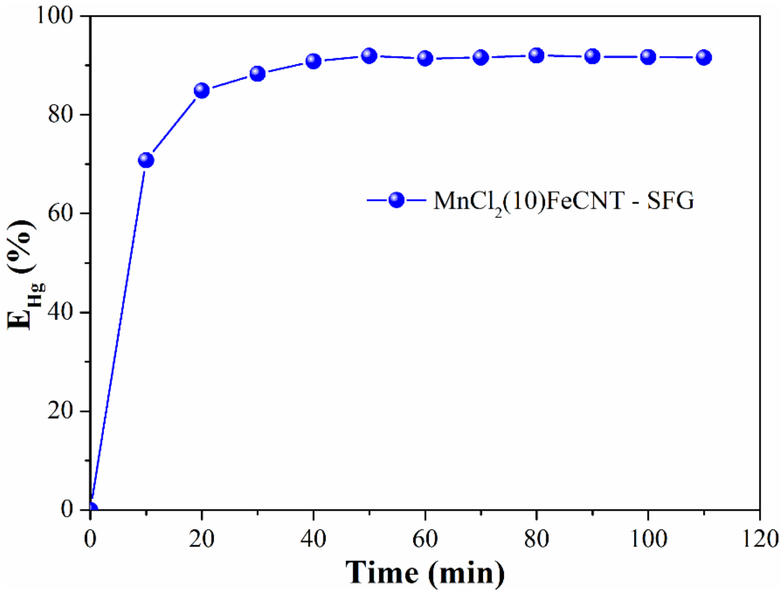

Figure S11 The regeneration performance of $MnCl_2$(10)FeCNT.

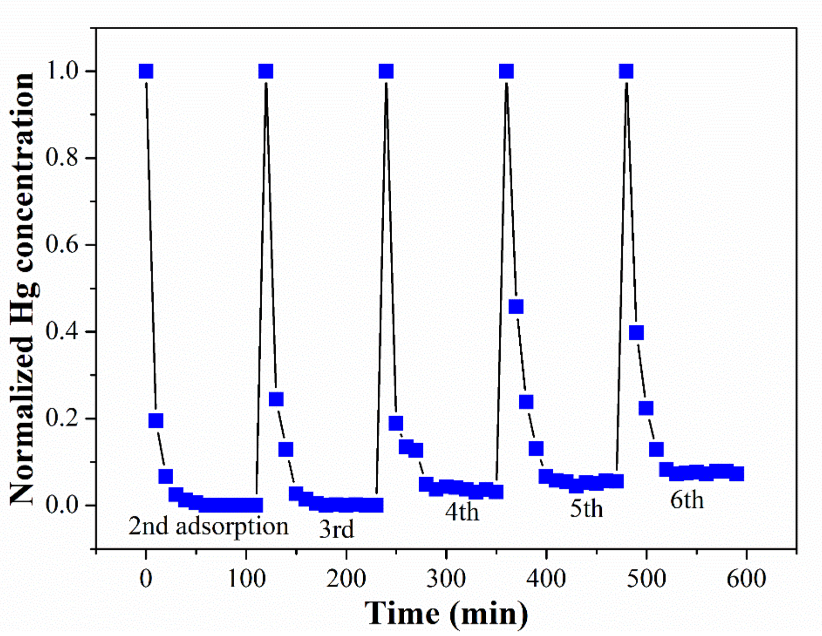

Figure S12 The XPS spectra pertaining to Mn 2p (a), Cl 2p (b), O 1s (c), and Fe 2p (d) of $MnCl_2$(10)FeCNT after the fifth regeneration; the N_2 adsorption/desorption curves (e) and pore size distribution curves (f) of $MnCl_2$(10)FeCNT after the fifth regeneration.

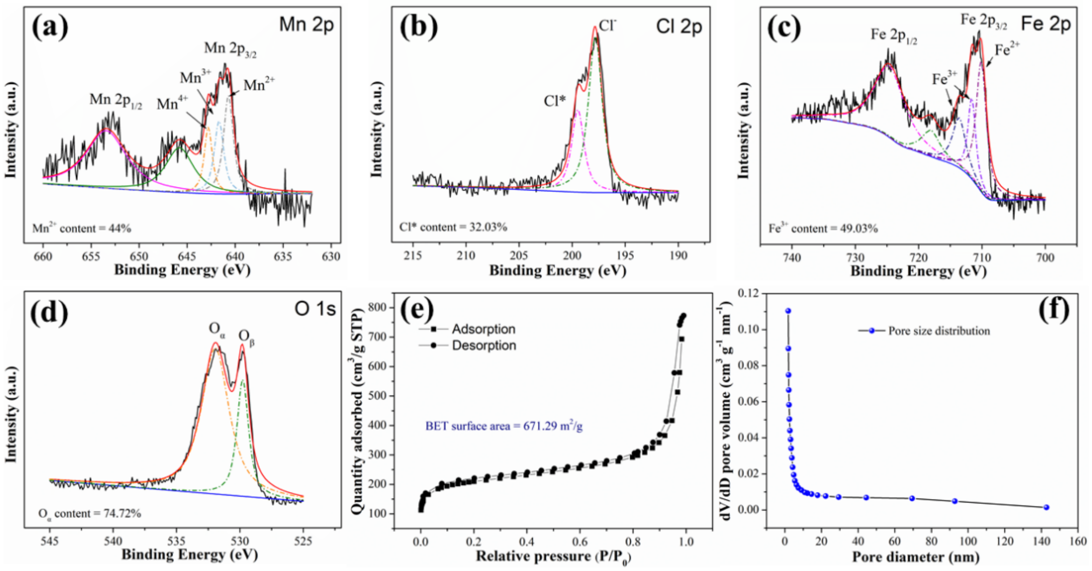

Table S1 The detailed experimental conditions for each experiment.

Experiments	Sample	Gas composition	Temperature
**Set 1.** Performance comparison	CNT/ FeCNT/ $MnCl_2$(10)FeCNT 100 mg	270 μg·m^{-3} Hg^0 N_2	100 °C
**Set 2.** Effect of $MnCl_2$ loading	$MnCl_2$-Fe_2O_3@CNT 1-15 wt% $MnCl_2$ 100 mg	270 μg·m^{-3} Hg^0 N_2	100 °C
**Set 3.** Effect of adsorption temperature	CNT $MnCl_2$(10)FeCNT 100 mg	270 μg·m^{-3} Hg^0 N_2	50-200 °C
**Set 4.** Effect of SO_2	CNT $MnCl_2$(10)FeCNT 100 mg	270 μg·m^{-3} Hg^0 0-1500 ppm SO_2 N_2	100 °C
**Set 5.** Effect of NO	CNT $MnCl_2$(10)FeCNT 100 mg	270 μg·m^{-3} Hg^0 0-800 ppm NO N_2	150 °C
**Set 6.** Effect of H_2O	CNT $MnCl_2$(10)FeCNT 100 mg	270 μg·m^{-3} Hg^0 0-6 %vol. H_2O N_2	150 °C
**Set 7.** Effects of HCl and NO_2	$MnCl_2$(10)FeCNT 100 mg	270 μg·m^{-3} Hg^0 50 ppm HCl 150-250 ppm NO_2 N_2	150 °C
**Set 8.** Effect of GHSV	$MnCl_2$(10)FeCNT 100 mg	270 μg·m^{-3} Hg^0 N_2	100 °C
**Set 9.** Performance under simulated flue gas	$MnCl_2$(10)FeCNT 100 mg	270 μg·m^{-3} Hg^0 5 %vol. O_2 500 ppm NO 1500 ppm SO_2 3 %vol. H_2O N_2	150 °C

Table S2 The comparison of $MnCl_2$(10)FeCNT with other reported sorbents.

**Sorbent**	**Hg^0 capture ability**	**Temperature**	**References**
AC-Br	2.3 $mg\cdot g^{-1}$	150 °C	[1]
Ag/graphene	2.4 $mg\cdot g^{-1}$ in 100 min	25 °C	[2]
$[MoS_4]^{2-}$/CoFe-LDH	16.39 $mg\cdot g^{-1}$	75 °C	[3]
CoMoS/γ-Al_2O_3	18.95 $mg\cdot g^{-1}$	50 °C	[4]
$LaMnO_3$	6.22 $mg\cdot g^{-1}$ in 600 min	150 °C	[5]
T6W1P1	0.353 $mg\cdot g^{-1}$	140 °C	[6]
RD-Cu_2S hollow nanocage	26.6 $mg\cdot g^{-1}$	120 °C	[7]
RPC-313	0.797 $mg\cdot g^{-1}$	120 °C	[8]
$Fe_3O_{4-x}Se_y$	8.8 $mg\cdot g^{-1}$	100 °C	[9]
SR_1T_7	9.63 $mg\cdot g^{-1}$	125 °C	[10]
CuS/CeO_2	32.304 $mg\cdot g^{-1}$	120 °C	[11]
Se/MIL-101	148.19 $mg\cdot g^{-1}$	100 °C	[12]
Ca_1-$Se_{1.7}$	1.806 $mg\cdot g^{-1}$	120 °C	[13]
MoS_x-6–220	53.03 $mg\cdot g^{-1}$	100 °C	[14]
$MnCl_2$(10)FeCNT	25.06 $mg\cdot g^{-1}$	100 °C	This work

Supplementary references

[1] Zhou Q, Duan Y, Hong Y, Zhu C, She M, Zhang J, et al. Experimental and kinetic studies of gas-phase mercury adsorption by raw and bromine modified activated carbon. Fuel Process Technol 2015; 134: 325-32.

[2] Xu H, Qu Z, Huang W, Mei J, Chen W, Zhao S, et al. Regenerable Ag/graphene sorbent for elemental mercury capture at ambient temperature. Colloids Surf, A 2015; 476: 83-9.

[3] Xu H, Yuan Y, Liao Y, Xie J, Qu Z, Shangguan W, et al. $[MoS_4]^{2-}$ cluster bridges in Co-Fe layered double hydroxides for mercury uptake from S-Hg mixed flue gas. Environ Sci Technol 2017; 51: 10109-16.

[4] Zhao H, Yang G, Gao X, Pang C, Kingman SW, Wu T. Hg^0 capture over CoMoS/γ-Al_2O_3 with MoS_2 nanosheets at low temperatures. Environ Sci Technol 2016; 50: 1056-64.

[5] Xu H, Qu Z, Zong C, Quan F, Mei J, Yan N. Catalytic oxidation and adsorption of Hg^0 over low-temperature NH_3-SCR $LaMnO_3$ perovskite oxide from flue gas. Appl Catal B: Environ 2016; 186: 30-40.

[6] Xu Y, Deng F, Pang Q, He S, Xu Y, Luo G, et al. Development of waste-derived sorbents from biomass and brominated flame retarded plastic for elemental mercury removal from coal-fired flue gas. Chem Eng J 2018; 350: 911-9.

[7] Wang L, Ji L, Li W, Zhang K, Xu H, Huang W, et al. Fabrication of Cu_2S hollow nanocages with enhanced high-temperature adsorption activity and recyclability for elemental mercury capture. Chem Eng J 2022; 427: 130935.

[8] Shi Q, Zhang X, Shen B, Ren K, Wang Y, Luo J. Enhanced elemental mercury removal via chlorine-based hierarchically porous biochar with $CaCO_3$ as template. Chem Eng J 2021; 406: 126828.

[9] Yang Z, Li H, Yang Q, Qu W, Zhao J, Feng Y, et al. Development of selenized magnetite ($Fe_3O_{4-x}Se_y$) as an efficient and recyclable trap for elemental mercury sequestration from coal combustion flue gas. Chem Eng J 2020; 394: 125022.

[10] Li H, LiLiu Q, Li J, Yang J, Long Y, Wang Y, et al. Removal of elemental mercury from flue gas over a low-cost and magnetic sorbent derived from $FeSO_4$-flocculated sludge and rice straw. J Energy Inst 2022; 105: 406-414.

[11] Xiao Y, Huang Y, Cheng H, Wu J, Jin B. Development of copper sulfide functionalized CeO_2 nanoparticle for strengthened removal of gaseous elemental mercury from flue gas. Chem Eng J 2023; 453: 139773.

[12] Yang J, Zhu W, Qu W, Yang Z, Wang J, Zhang M, et al. Selenium functionalized metal-organic framework MIL-101 for efficient and permanent sequestration of mercury. Environ Sci Technol 2019; 53: 2260-2268.

[13] Wang Y, Zhang Z, He C, He G, Zhang N, Zhang X, et al. Industrial grade calcium sulfide modified by selenium for elemental mercury removal from flue gas. Sep Purif Technol 2025; 354: 128632.

[14] Guan L, Chen Z, Liu Y, Wang R, Yan K, Xu Z, et al. Engineering sulfur-rich MoS_2 adsorbent with abundant unsaturated coordination sulfur sites for gaseous mercury capture from high-concentration SO_2 smelting flue gas. Chem Eng J 2024; 483: 149122.
